# Equilibrium Mechanics in Fracture Reduction of Tibial Plateau

**DOI:** 10.1111/os.70133

**Published:** 2025-08-18

**Authors:** Kaixuan Zhang, Xinrui Zhu, Wei Chen, Jing Na, Ningning Miao, Yuan Gao, Zhongzheng Wang, Yanbin Zhu, Shuang Yang, Yingze Zhang

**Affiliations:** ^1^ School of Medicine Nankai University Tianjin People's Republic of China; ^2^ Key Laboratory of Biomechanics of Hebei Province Orthopaedic Research Institute of Hebei Province Shijiazhuang People's Republic of China; ^3^ Department of Orthopedic Surgery of Hebei Province The Third Hospital of Hebei Medical University Shijiazhuang People's Republic of China; ^4^ National Health Commission (NHC) Key Laboratory of Intelligent Orthopeadic Equipment, The Third Hospital of Hebei Medical University Shijiazhuang People's Republic of China; ^5^ School of Mechanical Engineering Hebei University of Technology Tianjin People's Republic of China; ^6^ Chinese Academy of Engineering Beijing People's Republic of China

**Keywords:** double reverse traction repositor, equilibrium mechanics, homeopathic repositioning theory, minimally invasive therapy, tibial plateau fractures

## Abstract

**Background:**

Tibial plateau fracture is one of the common fractures in the lower limb, mostly caused by high‐energy injuries, which may be accompanied by different degrees of compression and displacement of the joint surface, affecting the knee joint alignment, stability, and sports function, and improper treatment may cause various complications, which are a more difficult problem in the clinic. The objective of this study was to investigate the biomechanical mechanisms underlying effective closed reduction in the treatment of tibial plateau fractures, particularly focusing on the performance of the homeopathic double reverse traction repositor compared to traditional traction table methods.

**Methods:**

We developed a biomechanical model to analyze the equilibrium mechanics during tibial plateau fracture reduction. A quantitative analysis was performed to evaluate the mechanical forces involved in both the traditional traction table method and the double reverse traction repositor.

**Results:**

Our analysis revealed that the use of a traction table generates an additional bending moment at the tibial plateau, resulting in medial over‐distraction and lateral compression. This mechanical imbalance can obstruct fracture reduction and irritate surrounding soft tissues. In contrast, the double reverse traction repositor avoids these adverse forces, reducing soft tissue irritation and improving reduction efficiency by utilizing equilibrium mechanics.

**Conclusion:**

The double reverse traction repositor offers a biomechanical advantage in the reduction of tibial plateau fractures by creating a more balanced mechanical environment. This study enhances the understanding of fracture reduction mechanics and supports the repositor's use not only for tibial plateau fractures but also for other fracture types, such as intertrochanteric, extremity long bone, and comminuted fractures.

## Graphical abstract

The theoretical analysis was established to study the equilibrium mechanics of tibial plateau fracture reduction, which confirms the advantages of using double reverse traction for tibial plateau fracture reduction compared with the traction table.

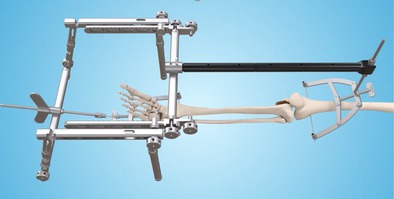

## 1 Introduction

Fractures have become a global public health problem as the number of people suffering from orthopedic diseases continues to increase [1, 2, 3, 4, 5, 6]. In 2019, there were about 455 million cases of fractures worldwide, a 70.1% increase since 1990. This rise not only affects the health status and quality of life of patients but also imposes a heavy burden on families and society [7, 8, 9]. Tibial plateau fractures (TPFs), as a typical type of closed fracture, account for approximately 1.66% of systemic fractures and around 27% of knee fractures [4]. Prompt anatomical reduction and fixation of TPFs reduce the risk of periarticular skin and soft tissue trauma and minimize the development of complications, both of which are essential for maintaining good knee function [10, 11, 12, 13, 14].

**FIGURE 1:**
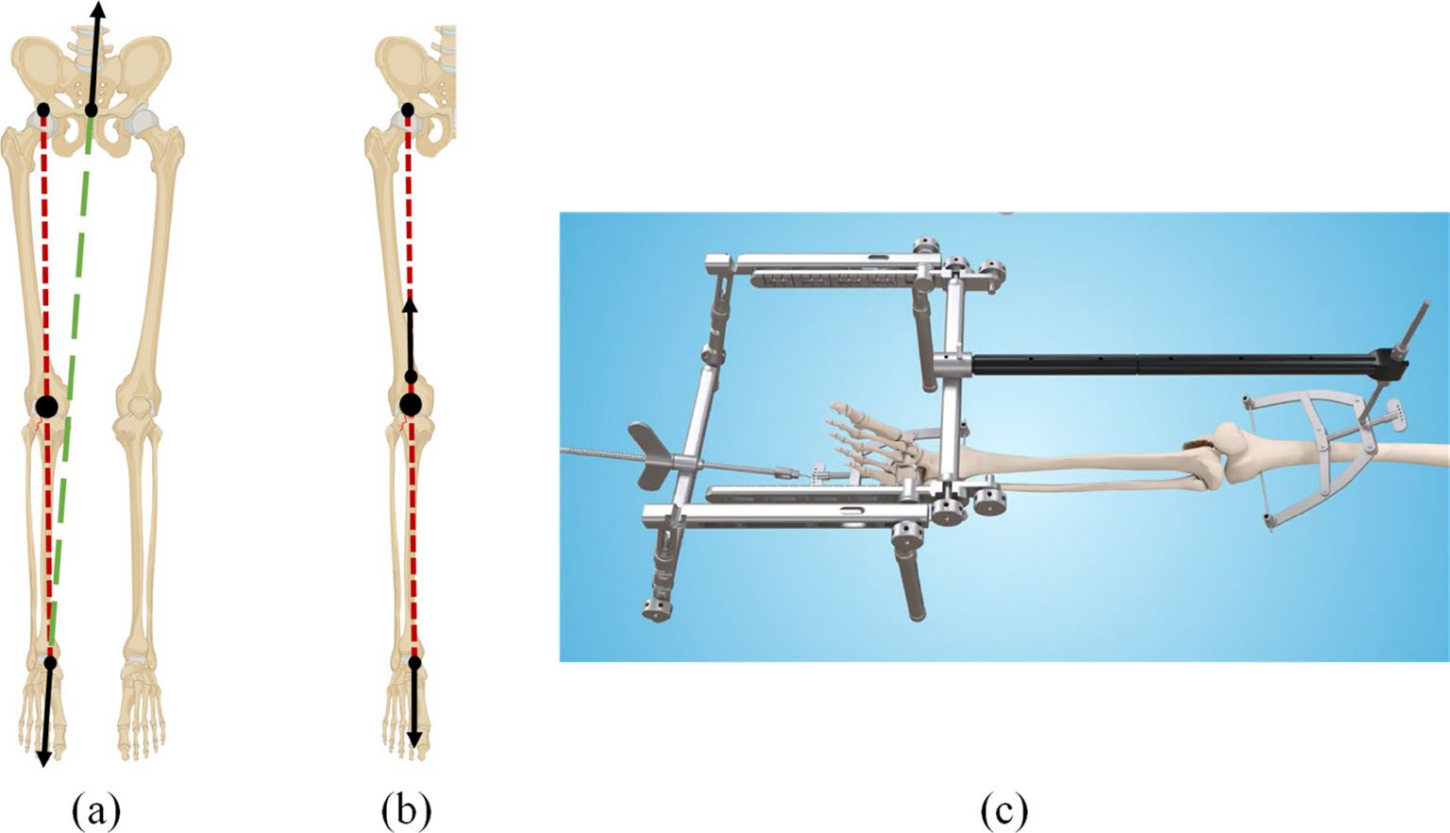
Schematic depicting (a) the mechanical axis of the lower limb (red short dash line) and the force line of the traction table (green long dashed line), illustrating the angle between them; (b) the force line of the DRTR aligned with the lower limb mechanical axis (red short dashed line); (c) illustration of minimally invasive therapy using the DRTR for tibial plateau fracture reduction [19].

The advent of modern medical technology has led to the gradual recognition of minimally invasive fracture treatment [15, 16, 17]. Traditional surgical reduction of fractures typically involves manipulation, traction table reduction, and femoral retractor reduction, commonly employed in clinical settings but also possessing inherent limitations [15]. In orthopedic traction tables, the most commonly utilized apparatus for reducing lower limb fractures, a notable biomechanical limitation exists [18]. The force line of the traction table and the mechanical axis of the lower limb form an angle during reduction, as shown in Figure 1a. This results in diminished traction force and a compromised reset effect, potentially leading to complications such as malrotation in femur stem fracture surgery [20, 21]. The positioning of the patient is critical when using an orthopedic traction table. The relatively intricate placement process, combined with extended preoperative preparation time, can precipitate complications during surgery, including incisional trauma to the perineum and surrounding tissue, damage to the sciatic and/or peroneal nerves, and a multitude of other potential injuries [22, 23].

The Association for the Study of Internal Fixation (AO/ASIF) has made significant advancements in the design and implementation of reduction tools and techniques for fracture treatment, leading to the development and adoption of minimally invasive procedures in orthopedic traumatology [24]. The design of the femoral retractor has revolutionized orthopedic surgery by transitioning from extracorporeal to intracorporeal traction. This shift has reduced the operator's workload and the need for intraoperative fluoroscopies, while also enhancing protection of the fracture site's blood circulation. Consequently, it promotes improved healing outcomes and reduces the risks of necrosis and infection [25]. However, the femoral retractor's traction capabilities are limited as they do not allow simultaneous adjustment in all three axes (X, Y, and Z), resulting in inconsistent traction forces that may compromise long‐term efficacy [26].

Yingze Zhang et al. [19, 27, 28, 29, 30] first proposed the theory of *homeopathic reduction*, integrating concepts from Chinese medicine such as simultaneous emphasis on tendon and bone, and a combination of movement and static principles. In line with this theory, the double reverse traction repositor (DRTR) was developed [31]. In the previous study of our research team, Wang et al. collected 187 consecutive adult patients with 189 operatively treated TPFs in the level I trauma center and all cases were performed by the senior surgeon using either the double reverse traction repositor or open reduction internal fixation. The results show that the double reverse traction repositor provides a safe reduction technique for the TPFs when used for the correct indications [28]. In addition, the traction force from the double reverse traction repositor was much larger than that from the table traction method [32]. During the procedure, auxiliary resetting pins are strategically placed at both ends of the fractured bone and connected to the double reverse traction bow to facilitate reverse bone traction. Figure 1b,c illustrate the schematic force lines and clinical animation of this process, respectively.

The traction repositor is designed to optimize soft tissue protection while maintaining blood supply to the fracture ends, thereby reducing trauma, complications, and promoting faster recovery and cost savings [33, 34]. Currently, the DRTR is widely adopted in minimally invasive lower limb fracture management, with numerous studies supporting its efficacy [35, 36, 37, 38, 39]. Ye et al. [40] investigated the biomechanics of long tubular bone fracture fixation using the DRTR, defining a reasonable range of fixation angles. Nonetheless, further investigation into the underlying mechanical principles of fracture reduction is warranted. The purpose of this study was to investigate the biomechanical mechanisms underlying effective closed reduction in the treatment of tibial plateau fractures, particularly focusing on the performance of the homeopathic double reverse traction repositor compared to traditional traction table methods.

## 2 Methods

### 2.1 Mechanical Analysis of Fracture Reduction With a Traction Table

**FIGURE 2:**
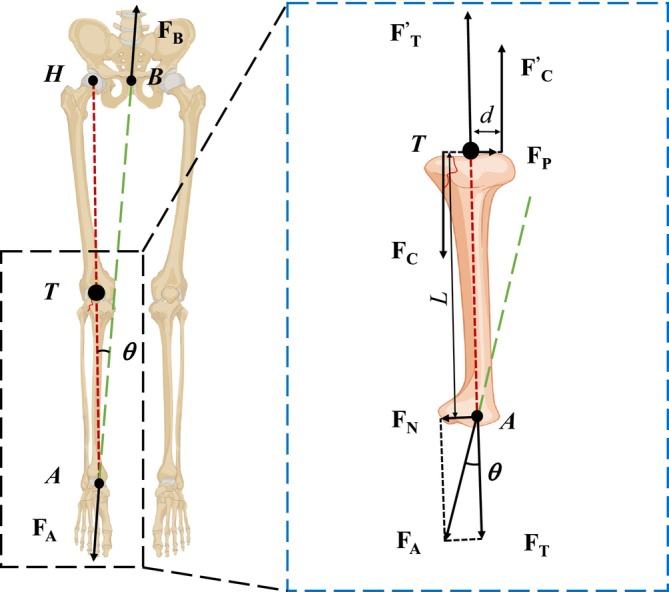
Equilibrium mechanical analysis of traction table reduction for tibial plateau fracture. θ is the angle between the traction force line and the mechanical axis of the lower limb.

Initially, tibial plateau fracture reduction was approached using a traction table. The mechanical analysis of the system involves considering a multi‐rigid body setup comprising skeletal components such as the hip, femur, tibia, and fibula. Assuming all forces act in a common plane, Figure 2 illustrates that traction force $F_{A}$ acts on the ankle from the hip center (Point B) towards the ankle (Point A). According to Newton's third law, the force $F_{B}$ acting on the hip is equal in magnitude but opposite in direction to $F_{A}$ when the entire system reaches equilibrium. The line of action of the traction force (green dashed line) forms an angle ($\theta \approx 8.8^{{}^{\circ}}$) with the lower extremity force line (red dashed line) from the hip (Point H) to the ankle (Point A). The detailed derivation of angle θ is shown in Figure S1 of the Supporting Information. According to the principles of equilibrium mechanics, when a multi‐rigid body system is in equilibrium, each constituent part must also be in equilibrium [41, 42]. Therefore, we can infer that the tibia itself is in equilibrium and analyze its mechanics accordingly, as depicted in the zoomed‐in section of Figure 2.

Due to the angle θ between the traction force line and the mechanical axis of the lower limb, the traction force at Point *A* can be decomposed into a component $F_{T}$ along the lower extremity force line and $F_{N}$ perpendicular to it. According to the principles of equilibrium and the force decomposition,

(1) $$\left\{\begin{array}{c} F_{T}=F_{A}\cdot \cos\theta \\ F_{N}=F_{A}\cdot \sin\theta \end{array}\right.$$

In clinical practice, $F_{T}$ aids in fracture reduction by providing direct bone‐to‐bone traction at the fracture site and indirect compression through surrounding soft tissues [17]. However, the presence of $F_{N}$, introduces a bending moment $M=F_{N}\cdot L$ and an additional equivalent shearing force $F_{P}$ from the surrounding tissues of the lower limb at reference point *T*. Here, *L* denotes the distance from traction point *A* to the referring point *T*. Additionally, there may be an equivalent tensile force $F_{T}^{\prime }$ and compressive force $F_{C}$ from the distal femur, creating a couple moment with an equivalent tensile force $F_{C}^{\prime }$ from surrounding soft tissues and a moment arm *d* at reference point *T*. The equilibrium conditions require that at any point, the combined force and bending moment on the system sum to zero,

(2) $$\left\{\begin{array}{c} F_{N}+F_{P}=0,F_{T}+F_{C}+F_{T}^{\prime }+F_{C}^{\prime }=0\;\\ M_{T}=F_{N}\cdot L+F_{C}\cdot d=0 \end{array}\right.$$

By combining Equations (1) and (2), we can derive the magnitudes of the additional shearing force $F_{P}$ and compressive force $F_{C}$ acting on the tibial plateau fracture,

(3) $$\left\{\begin{array}{c} F_{P}=F_{A}\cdot \sin\theta \\ F_{C}=F_{A}\cdot \frac{L}{d}\cdot \sin\theta \end{array}\right.$$

These two forces are generated by the traction force $F_{A}$ and can significantly disrupt fragment reduction during minimally invasive fixation and reduction.

### 2.2 Mechanical Analysis of Fracture Reduction With a Double Reverse Traction Repositor

Next, we consider tibial plateau fracture reduction using a DRTR. Unlike a traction table, where the upper traction force acts directly on the distal femur via a penetrating nail connected to the traction bow, we assume the multi‐rigid body system includes only the femur, tibia, and fibula. Additionally, all forces lie in the same plane, consistent with the use of a traction table.

**FIGURE 3:**
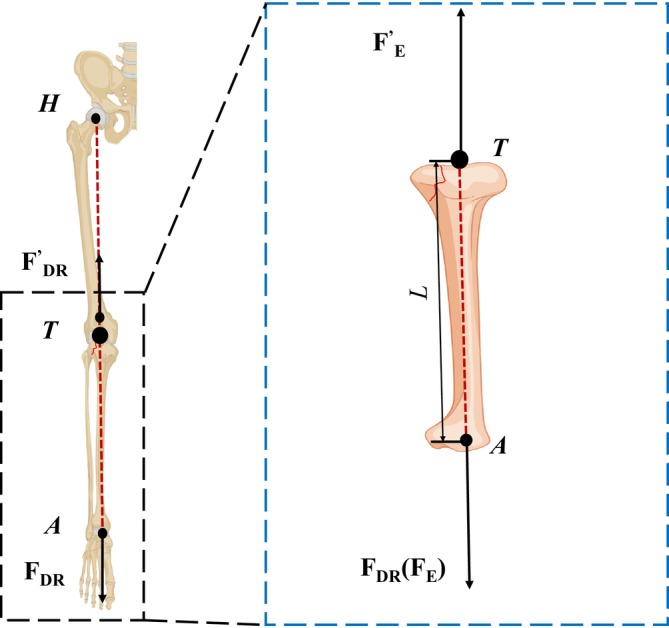
Equilibrium mechanical analysis of double reverse reduction for tibial plateau fractures.

In Figure 3, the multi‐body system reaches equilibrium under pairwise traction forces $F_{\mathrm{DR}}$ and $F_{\mathrm{DR}}^{\prime }$ generated by the DRTR. This allows for further analysis of the equilibrium mechanics of the tibia, as depicted in the zoomed‐in region of Figure 3. Since the direction of these traction forces aligns with the lower limb force line, the equilibrium condition can be expressed as

(4) $$F_{E}+F_{E}^{\prime }=0$$

in which $F_{E}=F_{\mathrm{DR}}$ represents the effective traction force exerted by the DRTR. $F_{E}^{\prime }$ denotes the bone‐to‐bone traction force from the distal femur, directly acting on the tibial plateau fracture due to the pairwise force $F_{\mathrm{DR}}^{\prime }$. This method ensures that the traction force applied to the fracture equals that produced by the DRTR, thereby enhancing the efficiency of fracture reduction. Importantly, it avoids additional shearing forces and bending moments, significantly reducing the agitation of the surrounding soft tissues and minimizing medically induced secondary injuries.

## 3 Results

### 3.1 Mechanical Response of Fracture Reduction With Different Traction Methods

The current DRTR uses the bone as the traction point to establish bone‐to‐bone traction, aligning the traction force with the mechanical axis of the lower extremity. In contrast, the traction table's force line forms an angle with the mechanical axis of the lower limb, resulting in unequal forces on the medial and lateral sides of the tibial plateau. This asymmetry prevents effective fracture repositioning and may lead to secondary damage to surrounding soft tissues.

**FIGURE 4:**
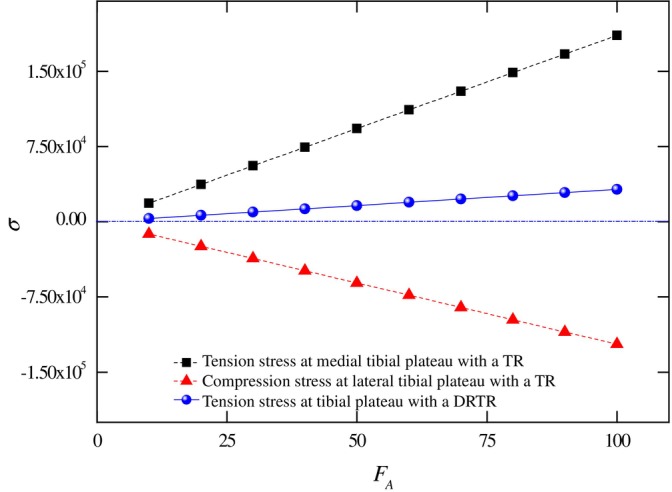
Stress distribution on the tibial plateau comparing a traction table repositor (TR) and a DRTR. Tension stress (dark square) at the medial tibial plateau and compression stress (red triangle) at the lateral tibial plateau under traction table use are shown, with stress values referenced to σ=0. Positive stress induced by the double reverse traction repositor is also depicted (blue circle).

Building on the equilibrium mechanics discussed above, we can further highlight the advantages of the DRTR over the traction table by analyzing local stress–strain in the tibial plateau. To simplify calculations, we approximate the tibial plateau as a rectangle with a width b=42.5±3.2 mm, and a height h=73±1.2 mm based on relevant experimental research [43]. The moment of inertia along the width side is $I_{z}=\frac{bh^{3}}{12}$. The maximum stress $\sigma_{\max}$ can be derived as $\sigma_{\max}=\frac{M}{W_{z}}$, where M is the bending moment and $W_{z}=2I_{z}/h$. The traction force from a traction table typically ranges from $F_{A}=50\sim 100$ N [17]. From Equation (1), the effective force along the mechanical axis of the lower limb $F_{T}$ ranges from 49.41 N to 98.82 N and the extra normal force $F_{N}$ ranges from 7.65 N to 15.3 N. This results in significant tensile and compressive stresses on the medial and lateral sides of the tibial plateau, respectively, under traction table use, as depicted in Figure 4. In contrast, the double reverse traction produces uniform tensile stresses without asymmetry between the two sides, even under the same traction force $F_{\mathrm{DR}}=F_{A}$. Furthermore, the maximum strain $\varepsilon_{\max}$ can be derived as $\varepsilon_{\max}=\sigma_{\max}/E$, where E is the modulus of the bone. For cortical bone with a modulus $E_{\mathrm{cor}}=12000$ MPa and cancellous bone with a modulus $E_{\mathrm{can}}=100$ MPa at the tibial plateau, the corresponding strain is calculated [44, 45].

### 3.2 The Effects of Osteoporosis on the Mechanical Response of Tibial Plateau

**FIGURE 5:**
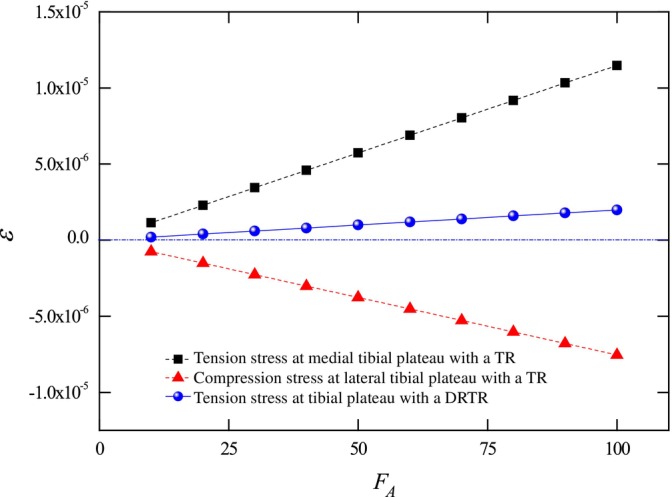
Strain distribution in the cortical bone of the tibial plateau comparing a traction table and a DRTR. Tension strain (dark square) at the medial tibial plateau and compression strain (red triangle) at the lateral tibial plateau under traction table use are shown, with strain values referenced to ε=0. Positive strain induced by the DRTR is also depicted (blue circle).

**FIGURE 6:**
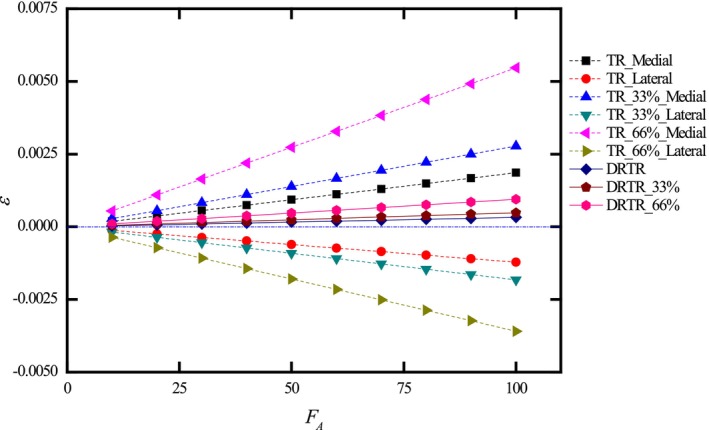
Strain distribution in the cancellous bone of the tibial plateau under various degrees of osteoporosis (0%, 33%, and 66% reduction in bone mass), comparing a traction table repositor (TR) and a double reverse traction repositor (DRTR). Tension strain at the medial tibial plateau (Medial) with a traction table repositor (TR) and compression strain at the lateral tibial plateau (Lateral) under traction table repositor (TR) use are shown, with strain values referenced to ε=0. Positive strain induced by the double reverse traction repositor (DRTR) is also depicted.

Considering osteoporosis, which affects elderly individuals, variations in bone elasticity are accounted for, reducing the modulus of cancellous bone by 33% ($E_{\mathrm{can}-33\%}=67$ MPa) and 66% ($E_{\mathrm{can}-66\%}=34$ MPa) for osteopenia and osteoporosis, respectively [45, 46]. The resulting strain in the tibial plateau under traction table and DRTR conditions is illustrated in Figures 5 and 6.

Figure 5 illustrates significant differences in strain induced by equivalent traction forces from a traction table and a DRTR, respectively. The traction table generates substantial tensile strain on the medial tibial plateau while also producing considerable compressive strain on the lateral tibial plateau. This imbalance not only hinders fracture reduction but also risks secondary damage to surrounding soft tissues, as discussed in the related clinical reports [21, 22, 35, 36, 38, 39, 47]. This limitation aligns with previous findings [17] on the restricted traction force achievable with traction tables.

## 4 Discussion

In this work, the equilibrium mechanics in fracture reduction of the tibial plateau with a double reverse traction repositor are provided systematically. Based on fundamental analysis, the DRTR can provide traction force which is along with the mechanical axis of the lower extremity compared to the traction table repositor. The clinical survey was conducted on patients with tibial plateau collapse fractures who received the novel bone graft reduction procedure with a bone tamp impactor instrument for minimally invasive treatment of tibial plateau collapse fractures in a tertiary referral university hospital from February 2021 to March 2023 [48]. Among patients with various types of tibial plateau fractures, over 60% achieved a postoperative articular surface depression depth of less than 2 mm, indicating precise reduction. Furthermore, the fracture fragment gap in all patients was less than 2 mm, with over 50% of patients across all tibial plateau fracture types achieving a postoperative gap of less than 1 mm, also demonstrating precise reduction. This technique offers several key advantages, summarized as follows: (1) Repositioning is aligned with the limb axis, ensuring consistency; (2) The repositioning force is robust, balanced, and continuous, aligning with natural physiological processes of soft tissue and bone growth; (3) Traction force is converted into squeezing and pushing at the proximal and distal ends, facilitating natural fracture repositioning; (4) The double reverse traction applies reciprocal forces to reposition the bone effectively; (5) Utilization of a double reverse traction bow enhances bone repositioning capabilities; (6) Repositioning follows anatomical principles. The traction repositor is designed to optimize soft tissue protection while maintaining blood supply to the fracture end, thereby reducing trauma, complications, and promoting faster recovery and cost savings. Currently, the DRTR is widely adopted in minimally invasive lower limb fracture management, supported by numerous studies demonstrating its efficacy.

Tibial plateau fractures are classified and treated based on the Schatzker classification [49]. Schatzker II–IV fractures are typically accompanied by varying degrees of widening of the tibial plateau and articular surface subsidence. Under continuous traction, the affected limb is typically suspended, with the traction force, rotational force, adduction, and abduction angles adjusted using a foot rotation rod, which may not align perfectly with the fracture line [50]. For fractures with anterior–posterior displacement, correction with traction alone is often inadequate. In such cases, open reduction is typically required, which may lead to extensive soft tissue damage, loss of blood supply to bone fragments, and increased risk of infection [51].

The DRTR achieves a uniformly lower strain across the tibial plateau, facilitating higher traction forces for fracture reduction and minimizing secondary injuries to surrounding soft tissues and blood vessels, which is consistent with clinical observations [17]. For elderly individuals with osteoporosis, as depicted in Figure 6, osteoporosis significantly exacerbates both tensile and compressive strain on the tibial plateau when using a traction table, further accentuating the disparity between the medial and lateral tibial plateau strains. Additionally, the operation of orthopedic traction beds is more complicated and requires longer preoperative preparation; intraoperative repositioning of the traction bed can easily lead to secondary injuries [52], and there is a higher incidence of postoperative complications due to the misalignment of the traction force with the machine's axis, such as perineal injuries.

Fractures from Schatzker I to III involve lateral tibial fractures, where the lateral collateral ligament and joint capsule are strained by traction. The lateral split fracture block is compressed and reduced, while the collapsed fracture block is reduced with top compression. A double traction frame is used to correct valgus deformity while maintaining alignment with the limb's mechanical axis. The tension from the ligaments and joint capsule, combined with a directional reduction rod, applies a reduction force to the collapsed bone fragments, while autologous iliac bone grafting supports the joint surface for effective reduction [53]. Schatzker IV involves a fracture of the medial condyle of the tibial plateau, where traction strains the medial collateral ligament and joint capsule, squeezing the medial fracture block into place. Schatzker V and VI are lateral tibial fractures, where the lateral collateral ligament and joint capsule are strained by traction. The lateral split fracture block is compressed and reduced, while the collapsed fracture block is reduced with top compression. For Schatzker IV to VI fractures, the double traction frame is more advantageous, as it provides stable and continuous traction while counteracting forces during reduction [53]. In the case of comminuted fractures, it is necessary to simplify the specific situation and establish an equivalent mechanical model to analyze the reduction behavior [54]. Rotating the distal DRTR handle generates traction force, improving rotational displacement, while overlapping displacement can be corrected by elevating the affected limb. Based on the theoretical analysis, further finite element analysis and in‐depth experimental studies will be conducted to enhance the study in future work.

There are still limitations of this study. The model we developed was based on bone‐to‐bone mechanical analysis in which we neglected the interaction of soft tissues and muscles around the tibial plateau. This may introduce some error into the conclusions. In the future, an improved model that includes soft tissue and periarticular muscles and ligaments could be considered. In addition, for comminuted fracture reduction, better theoretical models and reduction strategies need to be devised.

## 5 Conclusions

This paper investigates the equilibrium mechanics involved in tibial plateau fracture reduction through a comprehensive analysis of equilibrium theory. The findings reveal an inherent angle between the traction table's force line and the mechanical axis of the lower limb, which disrupts the physiological equilibrium at the tibial plateau. This misalignment limits the effectiveness of traction in fracture reduction and increases the risk of secondary injuries to surrounding soft tissues and blood vessels.

Furthermore, the local analysis of maximum stress and strain highlights the advantages of using double reverse traction for tibial plateau fracture reduction. Future studies could explore this method further by incorporating the structural characteristics of the tibial plateau and addressing the specific features of different fracture types. These insights may also provide a foundation for applying double reverse traction in the treatment of various orthopedic conditions, including intertrochanteric fractures, long bone fractures of the extremities, and comminuted fractures.

## Author Contributions

K.Z., X.Z., W.C., J.N., N.M., Y.G., Z.W., S.Y., and Y.Z. drafted the original manuscript and drew the figures. J.N., N.M., Z.W., Y.G., Y.Z., S.Y., and Y. Z. validated and revised the manuscript. K.Z., X.Z., W.C., S.Y., and Y.Z. developed the model and analyzed the data. W.C., S.Y., and Y.Z. designed and funded the project.

## Conflicts of Interest

The authors declare no conflicts of interest.

## Supporting information


**Data S1.** Supporting Information.
