# Supplementary material for: Equilibrium Mechanics in Fracture Reduction of Tibial Plateau
Source: Orthop Surg. 2025 Aug 18;17(10):2935–42. doi: 10.1111/os.70133 (PMC12497557; doi:10.1111/os.70133)
Supplement: Supplementary file 1 — Data S1. Supporting Information. [file OS-17-2935-s001.docx]

# Supplementary material for equilibrium mechanics in fracture reduction of tibial plateau

## The angle between the mechanical axis of lower limb and the traction force line of a traction table

We consider the tibial plateau fracture reduction by using a traction table. As shown in Fig. S1, the angle between the mechanical axis of the lower limb and the traction axis is $\theta$. As shown in the zoomed region in Fig. S1, based on the anatomy ^[1-5]^, the angle between the mechanical axis of the lower limb and the vertical axis is $\theta_{\alpha}=3^{^{\circ}}$, and the angle between the mechanical axis of the lower extremity and the anatomical axis of the femur is $\theta_{\beta}=6^{^{\circ}}$. $\theta_{f}={130}^{^{\circ}}\pm7^{^{\circ}}$ is the caput collum diaphyseal angle of the femur. From experimental results, we have the average length of femur (FT) $\mathrm{FT}=411.04\pm24.84$ mm, the average length of tibia (AT) $\mathrm{AT}=380\pm16.55$ mm, and the half distance between the centers of the hip joints on both sides $HB\approx100.0$ mm. We can obtain $\theta\approx{8.8}^{^{\circ}}$ from the geometric relationship. In this case, we can further obtain the traction force $\mathbf{F}_{T}$ and the extra perpendicular component force $\mathbf{F}_{N}$ from the traction table.

Figure S1. Geometric mechanics modeling based on human anatomy with a traction table. $\theta$ is the angle between the traction force line and the mechanical axis of the lower limb. $\theta_{\alpha}=3^{^{\circ}}$ is the angle between the mechanical axis of the lower limb and the vertical axis. $\theta_{\beta}=6^{^{\circ}}$ is the angle between the mechanical axis of the lower extremity and the anatomical axis of the femur. $\theta_{f}={130}^{^{\circ}}\pm7^{^{\circ}}$ is the caput collum diaphyseal angle of the femur

## References

[1] Marieb EN, Hoehn K. Human anatomy & physiology. Place: Pearson education; 2007.

[2] Zou L, Zhan C, Shi B, Zhuang J, Dong P, Zhu Y, et al. Measurement and clinical significance of geometric parameters of the knee joint in normal Chinese. Anatomy and Clinics 2010;15(4):243-46.

[3] Wang Y, Zhou F, Zhou Y, Cui J. Measurement and related research of the three-dimension morphology of the healthy Chinese knee. Orthopedic Journal of China 2004;12(8):617-19.

[4] Zhang Y. Clinical epidemiology of orthopaedic trauma. 3 ed., Place: People's Medical Publishing House; 2021.

[5] Yang Y, Du C, Lv H, Zhang Q, Chen W, Zhang Y. An imaging study of the relationship between tibiofibular length and height. Journal of Hebei Medical University 2014;35(11):1341-43.
